# XQueryer: an intelligent crystal structure identifier for powder X-ray diffraction

**DOI:** 10.1093/nsr/nwaf421

**Published:** 2025-09-30

**Authors:** Bin Cao, Zinan Zheng, Yang Liu, Longhan Zhang, Lawrence W-Y Wong, Lu-Tao Weng, Jia Li, Haoxiang Li, Tong-Yi Zhang

**Affiliations:** Guangzhou Municipal Key Laboratory of Materials Informatics, Hong Kong University of Science and Technology (Guangzhou), Guangzhou 511400, China; Advanced Materials Thrust, Function Hub, Hong Kong University of Science and Technology (Guangzhou), Guangzhou 511400, China; Data Science and Analytics Thrust, Information Hub, Hong Kong University of Science and Technology (Guangzhou), Guangzhou 511400, China; Data Science and Analytics Thrust, Information Hub, Hong Kong University of Science and Technology (Guangzhou), Guangzhou 511400, China; Hong Kong University of Science and Technology, Hong Kong 999077, China; Guangzhou Municipal Key Laboratory of Materials Informatics, Hong Kong University of Science and Technology (Guangzhou), Guangzhou 511400, China; Advanced Materials Thrust, Function Hub, Hong Kong University of Science and Technology (Guangzhou), Guangzhou 511400, China; Material Characterization and Preparation Facility, Hong Kong University of Science and Technology (Guangzhou), Guangzhou 511400, China; Guangzhou Municipal Key Laboratory of Materials Informatics, Hong Kong University of Science and Technology (Guangzhou), Guangzhou 511400, China; Material Characterization and Preparation Facility, Hong Kong University of Science and Technology (Guangzhou), Guangzhou 511400, China; Guangzhou Municipal Key Laboratory of Materials Informatics, Hong Kong University of Science and Technology (Guangzhou), Guangzhou 511400, China; Data Science and Analytics Thrust, Information Hub, Hong Kong University of Science and Technology (Guangzhou), Guangzhou 511400, China; Hong Kong University of Science and Technology, Hong Kong 999077, China; Guangzhou Municipal Key Laboratory of Materials Informatics, Hong Kong University of Science and Technology (Guangzhou), Guangzhou 511400, China; Advanced Materials Thrust, Function Hub, Hong Kong University of Science and Technology (Guangzhou), Guangzhou 511400, China; Guangzhou Municipal Key Laboratory of Materials Informatics, Hong Kong University of Science and Technology (Guangzhou), Guangzhou 511400, China; Advanced Materials Thrust, Function Hub, Hong Kong University of Science and Technology (Guangzhou), Guangzhou 511400, China; Materials Genome Institute, Shanghai Frontier Science Center of Mechanoinformatics, and Center for Integrated Circuits and Advanced Display Materials, Shanghai University, Shanghai 200444, China

**Keywords:** artificial intelligence, powder X-ray diffraction, crystal structure, intelligent identifier, XQueryer

## Abstract

Powder X-ray diffraction (PXRD) is a widely used technique for characterizing crystal structures. With the rise of artificial intelligence (AI) in science, the rapid expansion of AI laboratories and high-throughput characterization methods has created a growing demand for intelligent PXRD pattern analysis—that is, automated crystal structure identification in integrated AI-driven facilities. Such identifiers extract structural information directly from PXRD patterns and transmit it as accurate and efficient data streams to other intelligent components involved in material synthesis, characterization and optimization. In this work, we present XQueryer, an intelligent PXRD-based structure identifier. To support its development, we constructed a comprehensive simulation database using a newly designed high-fidelity simulation method. This database contains over two million PXRD patterns simulated from 100 315 crystal structures in the Materials Project, covering diverse intrinsic sample features and a range of extrinsic diffractometer conditions. XQueryer was evaluated on more than 200 000 simulated patterns and 1003 experimental PXRD patterns. It outperformed existing models and the traditional search–match method, achieving a 28.9% accuracy improvement compared with the second-best model. Furthermore, XQueryer has been integrated with a powder X-ray diffractometer, enabling real-time determination of crystal structures in tested samples.

## Teaser

We present XQueryer, an AI-powered system that identifies crystal structures from powder X-ray diffraction patterns, enabling faster and more reliable material discovery across different chemical elements.

## INTRODUCTION

**Figure 1.**
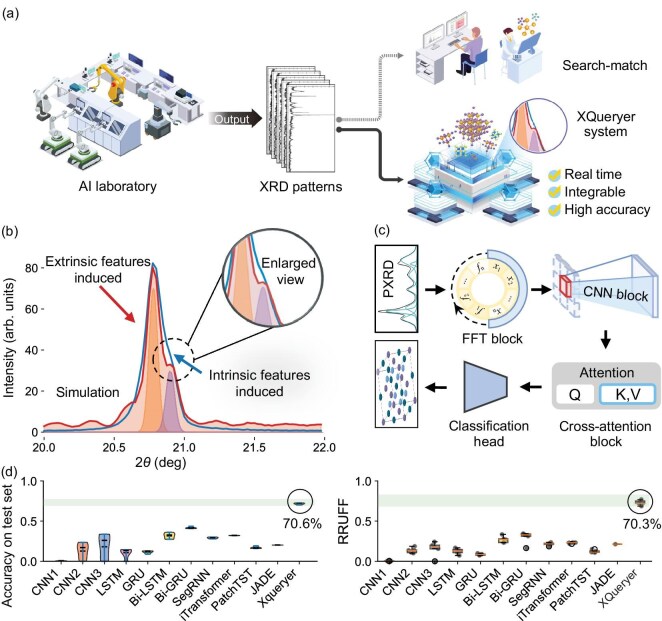
(a) Comparison between intelligent structure identification and traditional labor-intensive structure matching. (b) PXRD theory-guided simulations incorporate both intrinsic crystal features and extrinsic diffractometer factors for a given crystal and X-ray. (c) Architecture of the XQueryer network. (d) XQueryer achieves significantly higher accuracy on both simulated data and the experimental RRUFF 1003 dataset. A demonstration of the XQueryer system is available at https://www.youtube.com/watch?v=OYPoh7K5uM0.

Crystal structure characterization is fundamental in science, particularly in materials science and engineering. Among the available techniques, powder X-ray diffraction (PXRD) is especially powerful for revealing phase composition, lattice geometry and atomic positions. Owing to its sensitivity and element-specific scattering, each diffraction peak encodes critical information about a material’s atomic arrangement. Methodologies such as the Rietveld refinement [1], Pawley [2] and Le Bail methods [3] have enabled structure determination from PXRD patterns. However, despite these advances, many artificial-intelligence (AI)-driven laboratories [4–9] continue to generate vast amounts of PXRD data that cannot be analyzed quickly or efficiently. The traditional methods [10,11] remain labor-intensive and time-consuming, requiring manual adjustments, particularly for overlapping peaks, as illustrated in Fig. 1(a). This reliance on manual refinement limits throughput and prevents large-scale, automated analysis [10,12,13]. To meet growing demands, more intelligent solutions are needed. These should integrate PXRD theory, large crystal datasets, curated databases and modern AI techniques to accelerate and improve structural analysis. Yet, the scarcity of experimental data poses a major challenge. As of March 2024, the RRUFF database, one of the largest repositories of raw experimental PXRD patterns, contained 3002 inorganic entries [14]. After cleaning, only 1003 remained, which is far from sufficient to train deep neural networks (DNNs). High-fidelity, large-scale PXRD datasets are therefore critical, motivating the widespread use of simulated patterns in AI-based structure identification [15–23]. Several studies have leveraged simulated PXRD data to overcome the scarcity of experimental patterns. Park *et al.* [24] generated 150 000 inorganic crystal structure database (ICSD) patterns and achieved 81% space-group accuracy, while Chitturi *et al.* [25] trained on nearly one million ICSD/Cambridge structural database structures for lattice parameter prediction with $\sim$10% error. Building on this, Lee *et al.* [15,26] simulated millions of single- and multi-phase patterns from ICSD and MP, using convolutional neural networks, fully convolutional networks and transformers to reach $\sim$100% accuracy on experimental phase identification and $>$90% accuracy in crystal system prediction. Other strategies include CPICANN [22], which achieved up to 98.5% identification accuracy using crystallography open database (COD) data, and NPCNN [21], which reached 86% and 77% accuracy on the RRUFF crystal system and space-group classification, respectively. To mitigate dataset bias, Schopmans *et al.* [27] proposed on-the-fly generation of millions of patterns via symmetry operations, enabling ResNet training with $\sim$80% accuracy. In summary, simulated PXRD data have become essential for training AI models, with theory-based approaches providing scalable, accurate and physically grounded solutions for crystal structure identification.

In this paper, we present XQueryer, an intelligent crystal structure identifier for powder X-ray diffraction. We incorporate prior knowledge of PXRD theory to develop a simulation algorithm that accounts for diverse intrinsic sample features and various extrinsic characteristics of X-ray diffractometers. Using this algorithm, we generate 23 PXRD patterns per crystal, creating a high-fidelity simulated database of over two million entries from 100 315 crystal structures in the Materials Project database [28] (Fig. 1(b)). This simulated database significantly enhances XQueryer’s generalizability to experimental PXRD patterns, providing a domain-informed approach to data synthesis. XQueryer integrates Fourier transformation, convolutional neural networks (CNNs) and cross-attention blocks to achieve a deep and robust understanding of crystal structures in both real and reciprocal spaces. We evaluate its performance on more than 200 000 simulated and 1003 experimental PXRD patterns, comparing it against other AI-based PXRD models. XQueryer demonstrates high accuracy in crystal structure identification, outperforming both AI competitors and traditional search-matching methods on simulated and experimental datasets (Fig. 1(c)). Furthermore, XQueryer is integrated with the PANalytical Aeris Benchtop X-Ray diffractometer to form a software-hardware system capable of real-time analysis. This XQueryer system directly infers crystal structures and atomic positions from PXRD patterns within milliseconds (Fig. 1(d)), making it well suited for AI-driven laboratories.

## RESULTS

### Diffraction theory for high-fidelity pattern simulation

**Figure 2.**
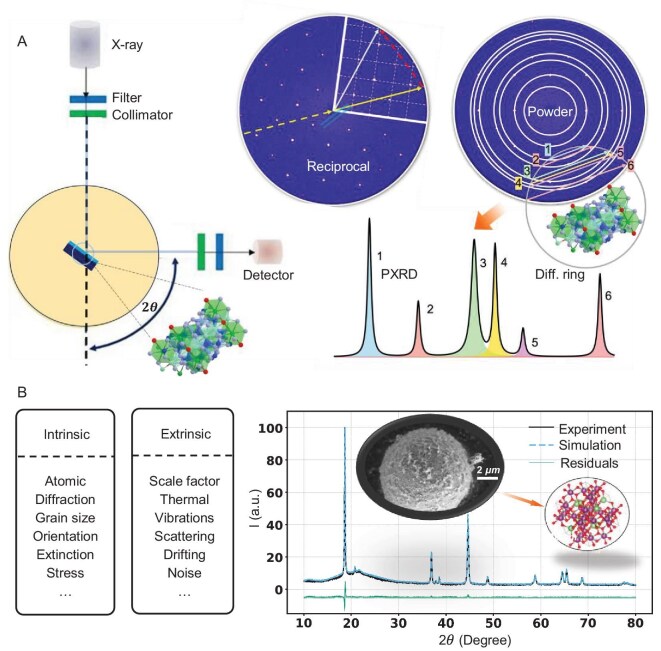
(a) Schematic of PXRD pattern generation for an experimental setup (left), showing the X-ray emitter, wavelength filter, incident beam on a powder sample, reflected beam and detector; a spot diffraction pattern of a single crystal in reciprocal space (middle) and a PXRD pattern derived from the ring diffraction pattern in reciprocal space (right). (b) Simulation workflow for a given crystal and X-ray wavelength: intrinsic and extrinsic feature values (left) are input into the simulation code to generate PXRD patterns, followed by comparison between simulated and experimental PXRD patterns of ${\rm Li}_{2}{\rm MnO}_{3}$ (right).

Figure 2(a) schematically illustrates the working principle of PXRD. When an incident X-ray beam strikes a sample at an angle $\theta$, it is elastically scattered, and the reflected beam is detected at the corresponding $2\theta$ angle by the PXRD diffractometer. X-ray diffraction arises from elastic interactions between X-ray photons and the electrons of atoms in the sample. Two key factors determine the fundamental diffraction pattern: an intrinsic feature—the atomic positions defining the crystal structure—and an extrinsic feature of the X-ray diffractometer. The middle panel of Fig. 2(a) illustrates this for a single crystal. X-ray diffraction effectively maps a crystal from real space to Fourier space, also known as reciprocal space. In PXRD, randomly oriented crystals in the powder convert discrete spot diffraction into ring-shaped diffraction, as shown in the final panel of Fig. 2(a). The PXRD diffractometer projects these three-dimensional diffraction signals onto the horizontal $2\theta$ axis, producing a PXRD pattern where the vertical axis represents diffraction intensity. Mathematically, a PXRD pattern can be described as a distribution function of diffraction intensity versus the 2$\theta$ angle.

There are diverse intrinsic sample features and various diffractometer extrinsic features that systematically affect the PXRD pattern, denoted by $ X_{\text{in}}$ and $ X_{\text{ex}}$, respectively. The interplay between intrinsic features $ X_{\text{in}}$ and extrinsic features $ X_{\text{ex}}$ is complex; coupling them can shift peak positions, alter peak shapes and generate background noise in PXRD patterns. Experimentally, a low-quality diffractometer typically produces high noise levels and significant peak overlap. The goal of AI-based PXRD models is to extract the intrinsic features $ X_{\text{in}}$ from diverse PXRD patterns influenced by both intrinsic and extrinsic factors. Our workflow for determining a crystal structure is divided into two tasks: first, identifying the reference crystal structure, and second, refining PXRD patterns to investigate the underlying physical mechanisms in XRD refinement. The present work focuses on the forward task. By fully considering both intrinsic features $ X_{\text{in}}$ and extrinsic features $ X_{\text{ex}}$, we simulate PXRD patterns using a crystal database retrieved from the Materials Project [28]. The dataset (MP-2024.1) contains 154 718 crystal entries as of January 2024. After removing duplicate or broken structures, 100 315 unique crystals remain. Figure 2(b) illustrates the simulation process. For each crystal and a given X-ray wavelength, we consider six intrinsic features ($ X_{\text{in}}$) and six extrinsic features ($ X_{\text{ex}}$). The intrinsic features include form factors, average grain size, deviation from random grain orientation, symmetry-related extinction effects, the Debye–Waller factor and internal stress. The extrinsic features include the scale factor, atomic thermal vibrations at room temperature, air scattering noise, instrumental vibrations, diffractometer setup effects represented by a convolution function and background noise. Because each feature can vary continuously, an infinite number of single-phase PXRD patterns can be generated for a given crystal and X-ray. In this work, we simulate PXRD patterns for each crystal by sampling from the feature space corresponding to the given crystals. Simulation details are provided in the Methods section below, and the simulation code is available in our PysimXRD package [29]. The right plot in Fig. 2(b) compares simulated and experimental PXRD patterns of ${\rm Li}_{2}{\rm MnO}_{3}$ at an X-ray wavelength of 1.540 56 Å, demonstrating excellent agreement [30]. Additional comparisons are presented in Figs S2 and S3, showing that varying feature values in simulations allows the PXRD patterns to encompass a wide range of experimental scenarios. This diversity enhances the generalizability of AI models trained on the large simulated dataset.

After performing high-throughput simulations, we constructed a comprehensive single-phase PXRD dataset. The dataset contains $100\, 315 \times 23$ PXRD patterns and is randomly divided into training and testing sets at a 21 : 2 ratio. Using the training set, we perform 21-fold cross-validation. It is worth noting that in-laboratory identification (see Section S4.2) is constrained by the coverage of the underlying crystal database. Expanding to a higher-quality database with broader structural diversity would enhance the practical applicability of this workflow.

### XQueryer demonstrates superior accuracy and generalizability

**Figure 3.**
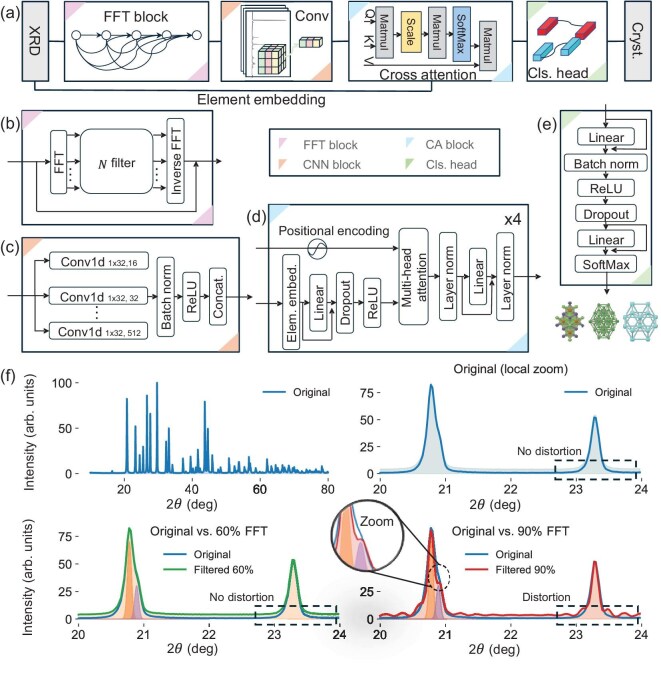
(a) Architecture of XQueryer, consisting of four main components: FFT block, CNN block, CA block and classification head. (b) The FFT block transforms the PXRD pattern into Fourier space, applies three filters (30%, 60% and 90%) to suppress high-frequency noise and then performs an inverse transform. The processed patterns are concatenated and passed to the CNN block. (c) The CNN block applies 1D kernels of varying sizes to capture features from different receptive fields; outputs are concatenated into tokenized sequences. (d) The CA block applies positional embedding to the feature sequence, employs Q-MLP layers for atomic embeddings and uses K-MLP and V-MLP layers for CNN-processed tokenized features. The five-head cross-attention blocks integrate crystal and pattern information. An atomic-information-free variant, XQueryer-NoElem, is implemented by zero-padding the atomic embeddings. (e) The classification head maps CA block outputs to crystal categories. (f) The FFT block reduces noise and peak overlap. For example, in the original PXRD pattern (2$\theta$ range: $20^\circ{\rm -}24^\circ$), overlapping peaks become distinguishable after FFT filtering at 60% and 90%.

In the present work, PXRD data were sampled at 3500 points over the 2$\theta$ range of $10^\circ$ to $80^\circ$. Figure 3(a) illustrates the XQueryer architecture, which comprises four main components: a fast Fourier transform (FFT) block, a CNN block, a cross-attention (CA) block and a classification head. The FFT block [31] combines FFT with a truncation filter, functioning as a low-frequency-pass filter. This reduces noise and peak overlap (Fig. 3(f)) caused by scattering and photoelectron effects during diffraction [32], while overly aggressive filtering may obscure fine peak details. The FFT converts the 3500 PXRD points (2$\theta$, intensity) into frequency–amplitude pairs. A filter ratio, defined as the fraction of high-frequency components relative to the total frequency range, is applied at three levels: 30%, 60% and 90%. The filtered data are then transformed back into the original space using the inverse FFT. To prevent loss of peak information in the original PXRD patterns, a residual connection is applied, as shown in Fig. 3(b). The filtered PXRD signals are concatenated with the original signal and then fed into the CNN block. In each CNN channel, one-dimensional (1D) convolution with various kernel sizes ($n\times 1$) captures peak shapes through multi-layer convolution. In the cross-attention setup [33], elemental information from the atoms in a crystal serves as the query to refine pattern features. Atomic information is embedded using the same feature set as in the CGCNN [34] and then processed by Q-MLP layers to generate the query vector (Q vector), which is fed into the cross-attention module. The main input to the CA block is the tokenized CNN output, with positional encoding applied. The token embeddings pass through K-MLP and V-MLP layers to generate key (K) and value (V) vectors. The cross-attention module then integrates chemical information and extracts structural insights from the PXRD patterns. Inspired by BERT [35], the classification head deciphers the first-token input to one of 100 315 crystal types. Detailed ablation studies are provided in Table S1.

The performance of XQueryer is evaluated on both a simulated testing dataset and experimental PXRD data from RRUFF [14]. For a fair comparison, we also include results for XQueryer-NoElem, which employs the original XQueryer architecture (Fig. 3) with element information masked via zero-padding. These results are compared with those obtained using the line-based search-and-match algorithm from the JADE Pro 8.9 software’s Task-Macro module [36]. Eight published CNN-based models for PXRD [15,17–19,24,26,37,38] are categorized by the number of convolutional and pooling layers, as well as the use of ensemble learning and dropout techniques. Additionally, eleven other AI algorithms designed for sequential data analysis are employed, including RNN [39] LSTM [40], GRU [41], BiRNN, BiLSTM, BiGRU [42], DLinear [40], SegRNN [40], the vanilla Transformer model [43], iTransformer [44] and PatchTST [45]. Performance tests are conducted on three tasks: structure identification, crystal system classification and space group classification. The structure identification task evaluates whether each of the more than 200 000 simulated PXRD patterns or 1003 experimental patterns can be correctly assigned to one of 100 315 crystal types. Similarly, the crystal system and space group classification tasks assess whether these patterns can be accurately classified into one of seven crystal systems or one of 230 space groups, respectively. The test results indicate that most of the aforementioned models perform effectively in crystal system and space group classification. CNNs without pooling layers (CNN3) outperform others, as pooling can lead to information loss. Bidirectional recurrent models consistently surpass their unidirectional counterparts. However, all these models struggle with structure identification. In particular, the five CNN models, the RNN and BiRNN models, the vanilla Transformer model and the DLinear model fail to perform effectively in this task. Therefore, Table 1 lists the performances of CNN1, CNN2, CNN3, LSTM, GRU, BiLSTM, BiGRU, SegRNN, iTransformer and PatchTST, alongside the XQueryer, XQueryer-NoElem models, and the JADE Pro 8.9 method.

**Table 1.** Prediction accuracy, F1 score, precision and recall for structure identification, crystal system classification and space group classification across different models on simulated and experimental test data.

	Baselines				Simulation				RRUFF			
Model	Conv.	Pooling	Ensemble	Ref.	Accuracy	F1	Precision	Recall	Accuracy	F1	Precision	Recall
**Structure identification**												
CNN1	6	MaxPool	$\checkmark$	[17]	0.005$\pm$0.001	0.001$\pm$0.001	0.001$\pm$0.001	0.005$\pm$0.001	0.003$\pm$.001	0.001$\pm$0.001	0.001$\pm$0.001	0.002$\pm$0.001
CNN2	4	MaxPool	$\times$	[38]	0.126$\pm$0.099	0.111$\pm$0.088	0.123$\pm$0.097	0.126$\pm$0.099	0.134$\pm$0.034	0.074$\pm$0.020	0.073$\pm$0.019	0.076$\pm$0.020
CNN3	3	None	$\times$	[21]	0.180$\pm$0.166	0.162$\pm$0.151	0.171$\pm$0.159	0.180$\pm$0.166	0.155$\pm$0.092	0.088$\pm$0.053	0.088$\pm$0.053	0.090$\pm$0.054
LSTM					0.106$\pm$0.060	0.079$\pm$0.045	0.078$\pm$0.045	0.106$\pm$0.060	0.124$\pm$0.036	0.067$\pm$0.021	0.067$\pm$0.021	0.069$\pm$0.021
GRU					0.115$\pm$0.017	0.082$\pm$0.015	0.080$\pm$0.015	0.115$\pm$0.017	0.084$\pm$0.010	0.044$\pm$0.005	0.044$\pm$0.006	0.046$\pm$0.005
Bidirectional-LSTM					0.315$\pm$0.037	0.284$\pm$0.037	0.296$\pm$0.040	0.315$\pm$0.037	0.270$\pm$0.041	0.160$\pm$0.028	0.159$\pm$0.028	0.162$\pm$0.029
Bidirectional-GRU					0.416$\pm$0.011	0.381$\pm$0.011	0.397$\pm$0.012	0.416$\pm$0.011	0.297$\pm$0.075	0.179$\pm$0.050	0.178$\pm$0.049	0.183$\pm$0.050
SegRNN					0.290$\pm$0.009	0.256$\pm$0.008	0.280$\pm$0.008	0.290$\pm$0.009	0.213$\pm$0.018	0.123$\pm$0.012	0.121$\pm$0.012	0.126$\pm$0.012
iTransformer					0.321$\pm$0.002	0.294$\pm$0.002	0.310$\pm$0.003	0.321$\pm$0.002	0.229$\pm$0.007	0.132$\pm$0.004	0.131$\pm$0.004	0.134$\pm$0.004
PatchTST					0.169$\pm$0.013	0.145$\pm$0.012	0.160$\pm$0.013	0.169$\pm$0.013	0.121$\pm$0.017	0.067$\pm$0.010	0.066$\pm$0.010	0.069$\pm$0.010
JADE Pro 8.9					0.212	–	–	–	0.226	–	–	–
XQueryer					0.705$\pm$0.017	0.704$\pm$0.012	0.686$\pm$0.023	0.708$\pm$0.018	0.703$\pm$0.031	0.698$\pm$0.031	0.629$\pm$0.041	0.650$\pm$0.020
XQueryer-NoElem					0.654$\pm$0.011	0.627$\pm$0.031	0.646$\pm$0.013	0.654$\pm$0.004	0.515$\pm$0.021	0.526$\pm$0.013	0.531$\pm$0.013	0.519$\pm$0.026
**Crystal system classification**												
CNN1	6	MaxPool	$\checkmark$	[17]	0.300$\pm$0.017	0.241$\pm$0.022	0.238$\pm$0.019	0.253$\pm$0.023	0.290$\pm$0.021	0.262$\pm$0.020	0.280$\pm$0.020	0.263$\pm$0.020
CNN2	4	MaxPool	$\times$	[38]	0.462$\pm$0.203	0.410$\pm$0.234	0.427$\pm$0.252	0.426$\pm$0.183	0.483$\pm$0.203	0.438$\pm$0.245	0.476$\pm$0.274	0.447$\pm$0.193
CNN3	3	None	$\times$	[21]	0.510$\pm$0.217	0.431$\pm$0.307	0.430$\pm$0.323	0.460$\pm$0.260	0.508$\pm$0.250	0.441$\pm$0.321	0.454$\pm$0.346	0.466$\pm$0.266
LSTM					0.451$\pm$0.160	0.431$\pm$0.171	0.445$\pm$0.151	0.447$\pm$0.138	0.457$\pm$0.156	0.440$\pm$0.167	0.480$\pm$0.141	0.444$\pm$0.134
GRU					0.478$\pm$0.023	0.448$\pm$0.028	0.449$\pm$0.028	0.450$\pm$0.029	0.479$\pm$0.032	0.461$\pm$0.034	0.483$\pm$0.038	0.453$\pm$0.032
Bidirectional-LSTM					0.706$\pm$0.024	0.698$\pm$0.023	0.700$\pm$0.023	0.697$\pm$0.023	0.721$\pm$0.026	0.719$\pm$0.022	0.735$\pm$0.022	0.708$\pm$0.022
Bidirectional-GRU					0.773$\pm$0.008	0.765$\pm$0.009	0.767$\pm$0.009	0.763$\pm$0.010	0.769$\pm$0.018	0.771$\pm$0.019	0.779$\pm$0.020	0.764$\pm$0.019
SegRNN					0.686$\pm$0.007	0.672$\pm$0.007	0.670$\pm$0.008	0.675$\pm$0.006	0.694$\pm$0.009	0.695$\pm$0.008	0.710$\pm$0.008	0.683$\pm$0.008
iTransformer					0.683$\pm$0.002	0.660$\pm$0.003	0.663$\pm$0.001	0.659$\pm$0.004	0.693$\pm$0.003	0.690$\pm$0.004	0.711$\pm$0.004	0.676$\pm$0.005
PatchTST					0.575$\pm$0.011	0.557$\pm$0.011	0.548$\pm$0.013	0.568$\pm$0.010	0.592$\pm$0.008	0.586$\pm$0.010	0.598$\pm$0.011	0.579$\pm$0.009
JADE Pro 8.9					0.706	–	–	–	0.747	–	–	–
XQueryer		0.936$\pm$0.011	0.923$\pm$0.013	0.921$\pm$ 0.008	0.926$\pm$0.017	0.933$\pm$0.016	0.920$\pm$0.011	0.928$\pm$0.021	0.924$\pm$0.013			
XQueryer-NoElem					0.913$\pm$0.021	0.898$\pm$0.015	0.900$\pm$0.015	0.895$\pm$0.011	0.915$\pm$0.015	0.905$\pm$0.020	0.912$\pm$0.007	0.899$\pm$0.004
**Space group classification**												
CNN1	6	MaxPool	$\checkmark$	[17]	0.122$\pm$0.014	0.028$\pm$0.003	0.041$\pm$0.008	0.038$\pm$0.007	0.113$\pm$0.106	0.044$\pm$0.002	0.059$\pm$0.007	0.047$\pm$0.005
CNN2	4	MaxPool	$\times$	[38]	0.308$\pm$0.191	0.244$\pm$0.167	0.270$\pm$0.178	0.245$\pm$0.163	0.326$\pm$0.199	0.198$\pm$0.131	0.221$\pm$0.142	0.207$\pm$0.133
CNN3	3	None	$\times$	[21]	0.376$\pm$0.240	0.299$\pm$0.245	0.305$\pm$0.250	0.302$\pm$0.245	0.375$\pm$0.270	0.260$\pm$0.213	0.272$\pm$0.223	0.274$\pm$0.220
LSTM					0.286$\pm$0.126	0.198$\pm$0.100	0.199$\pm$0.100	0.213$\pm$0.105	0.288$\pm$0.132	0.165$\pm$0.082	0.182$\pm$0.089	0.172$\pm$0.081
GRU					0.307$\pm$0.024	0.195$\pm$0.025	0.197$\pm$0.025	0.208$\pm$0.025	0.324$\pm$0.035	0.171$\pm$0.028	0.195$\pm$0.035	0.176$\pm$0.030
Bidirectional-LSTM					0.568$\pm$0.035	0.489$\pm$0.043	0.488$\pm$0.045	0.499$\pm$0.040	0.587$\pm$0.027	0.407$\pm$0.030	0.425$\pm$0.029	0.425$\pm$0.033
Bidirectional-GRU					0.664$\pm$0.012	0.612$\pm$0.017	0.621$\pm$0.018	0.615$\pm$0.017	0.663$\pm$0.018	0.495$\pm$0.020	0.514$\pm$0.022	0.506$\pm$0.018
SegRNN					0.552$\pm$0.009	0.495$\pm$0.013	0.506$\pm$0.011	0.502$\pm$0.015	0.566$\pm$0.013	0.388$\pm$0.023	0.414$\pm$0.022	0.397$\pm$0.026
iTransformer					0.575$\pm$0.003	0.496$\pm$0.002	0.502$\pm$0.002	0.501$\pm$0.003	0.584$\pm$0.010	0.397$\pm$0.010	0.423$\pm$0.010	0.406$\pm$0.014
PatchTST					0.425$\pm$0.013	0.334$\pm$0.019	0.346$\pm$0.020	0.340$\pm$0.019	0.455$\pm$0.015	0.267$\pm$0.023	0.293$\pm$0.020	0.273$\pm$0.029
JADE Pro 8.9					0.604	–	–	–	0.597	–	–	–
XQueryer					0.894$\pm$0.012	0.867$\pm$0.011	0.872$\pm$ 0.011	0.865$\pm$0.019	0.888$\pm$0.012	0.828$\pm$0.012	0.843$\pm$0.018	0.837$\pm$0.020
XQueryer-NoElem					0.863$\pm$0.017	0.828$\pm$0.012	0.837$\pm$0.011	0.823$\pm$0.015	0.878$\pm$0.013	0.738$\pm$0.016	0.752$\pm$0.008	0.754$\pm$0.011

The comparison of model performance on three tasks, listed in Table 1 and Fig. S1, demonstrates that XQueryer outperforms the second-best model on the simulation test set by 28.9% in accuracy, 32.3% in F1, 28.9% in precision and 29.2% in recall for structure identification. On the experimental test set, XQueryer achieves 70.3% accuracy, 69.8% F1, 62.9% precision and 65.0% recall, surpassing the second-best model in all metrics. XQueryer leads in all tasks, with improvements of 47.7% (structure identification), 18.6% (crystal system classification) and 29.1% (space group classification) over the search-match strategy on the experimental RRUFF database. This superior performance is largely attributed to its training on the largest simulated PXRD dataset to date, which grants strong generalizability across diverse chemical systems.

To further assess its capability, XQueryer was evaluated against published AI models whose simulated or experimental PXRD datasets are publicly available. In this comparison, XQueryer was applied directly, without additional fine-tuning, to these open datasets, while the reported metrics of the published models were taken from their respective papers. Table 2 shows that XQueryer and XQueryer-NoElem outperform other models across most open test sets, except CNN1’s, where CNN1 achieves higher accuracy in structure identification. This gap arises from differences in crystal coverage between the ICSD and MP databases. When crystals lie outside MP’s scope, direct structure identification becomes challenging. Overall, the results highlight the strong performance of the XQueryer architecture, particularly on experimental datasets. Notably, even without element information, it consistently surpasses other models under general conditions.

**Table 2.** Comparison of models on published test datasets. The comparison includes crystal source (source), number of crystals (NCryst.), test dataset size (TSize), data types (types), task setting (tasks) aligned with our Table 1, author-reported accuracy (rep.), XQueryer accuracy (XQ) and XQueryer-NoElem accuracy (NoElem).

Model	Source	NCryst.	TSize	Type	Task	Rep. (%)	XQ (%)	NoElem (%)
CNN1 [17]	ICSD	140	4200	Sim.	Structure	94.0	82.0	71.0
CNN3 [21]	ICSD & MP	171 006	2253	Sim.	Space group	45.0	87.0	71.9
					Crystal system	75.0	94.0	81.9
CPICANN [22]	COD	23 073	100	Expt.	Structure	80.0	97.0	94.0
ResNet-50 [27]	ICSD	272 260	1003	Expt.	Space group	25.2	88.7	86.3

### FFT block enhances the effectiveness of structure identification

**Figure 4.**
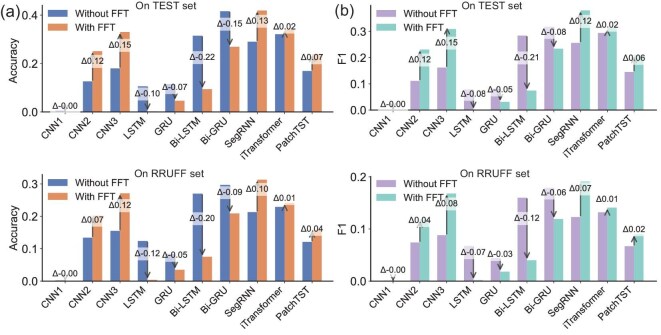
The performance of models on structure identification with and without the FFT block. (a) Comparison of classification accuracy with and without the FFT block on the simulated test dataset and the experimental dataset. (b) Comparison of the F1 score on the simulated test dataset and the experimental dataset. Positive values indicate an improvement, while negative values indicate a decrease.

The FFT block greatly reduces noise by transforming diffraction signals from the real PXRD domain to the frequency domain. Figure 3(f) indicates that the overlapping peaks are split with filtering ratios of 60% and 90%. Noise reduction is especially important in PXRD because both intrinsic and extrinsic features contribute to noise. To assess the impact of the FFT block, we compare the performance of several DNN models on crystal structure identification, with and without the FFT block, using both simulated and experimental PXRD datasets and plot the results in Fig. 4, where the performance is represented by the metrics of accuracy and the F1 score.

Figure 4  shows the comparison of accuracy and F1 score on both the simulated and RRUFF datasets, indicating that the FFT block significantly improves performance. The CNN2’s accuracy improves from 0.126 to 0.251, and the CNN3’s from 0.180 to 0.330. For the iTransformer model, the F1 score increases from 0.229 to 0.241. Figure 4 also illustrates the comparison on experimental RRUFF data, where CNN2 and SegRNN show accuracy gains from 0.134 to 0.202 and 0.213 to 0.313, respectively; iTransformer increases from 0.229 to 0.241 in accuracy, along with improvements in the F1 score. These consistent gains highlight the FFT block’s effectiveness in handling noisy PXRD patterns. The FFT block, however, does not yield significant improvements for sequential models such as LSTM and GRU.

To further investigate the FFT block’s effect on patterns with varying peak widths, we divide the dataset into two subsets: one with narrow peak distributions (average grain size $\ge$70 nm, thermal vibration $ \le$0.05 Å, detector height $ \le$10 mm), and one with broad peak distributions (all other samples). We then compare classification accuracy with and without the FFT block. The results, summarized in Table S3, indicate a generally consistent trend across both subsets: the FFT block enhances model performance or causes minimal degradation. However, the performance gain is typically larger for the broad peak subset. This suggests that the FFT block is particularly helpful in handling broadened peaks, likely due to its ability to mitigate peak overlapping, as illustrated in Fig. 3(f).

### An integrated software-hardware smart system enables real-time crystal analysis

**Figure 5.**
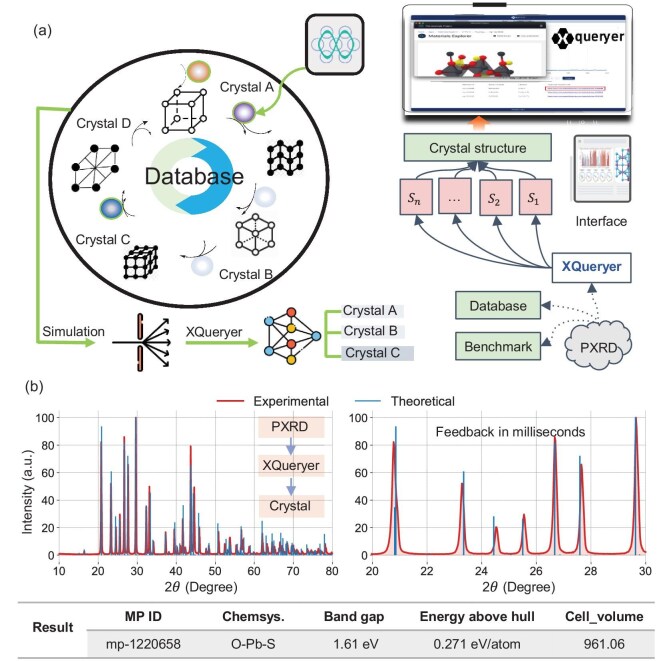
The intelligent PXRD structure identification process in the XQueryer system (https://www.youtube.com/watch?v=OYPoh7K5uM0). (a) The XQueryer system comprises several key components: a high-fidelity PXRD data generation pathway, a comparison benchmark (detailed in Table 1), an excellent identification model, an open-access, multi-site website and an integrated, automated characterization framework. (b) The XQueryer system enables the real-time identification of raw experimental PXRD data and provides comprehensive information on crystal structures and physical attributes.

We integrate the XQueryer model with the PANalytical Aeris Benchtop X-Ray diffractometer [46]. This diffractometer supports third-party software installation, facilitating a seamless connection between the two systems. The integration is made possible through a portable software module that, upon defining the root directory, allows XQueryer to automatically detect the file root generated by the diffractometer. Once the machine completes a scan and generates a new XPD file, the XQueryer model is triggered to read the corresponding XRDML file. The software then parses the file into a standardized PXRD sequence for crystal phase identification. To ensure smooth operation, the integration is designed with error-handling capabilities, such as file integrity checks and real-time feedback on processing status. The parsed results, including phase identification and analysis data, are automatically saved in the same folder as the raw XRD data. This streamlined workflow enables the efficient and automated acquisition of crystal structures from powder samples without manual intervention, as demonstrated in Fig. 5. Additionally, the system’s modular design allows future upgrades to incorporate additional file formats or data processing techniques. The detailed decomposition steps are provided in Section S4.6.

Starting from a powder sample, the diffractometer collects PXRD data, while the XQueryer model identifies crystal structures and provides recommendations with a direct link to the MP detail page. All structures and physical properties retrieved from the MP database are provided as supplementary material. These are automatically exported locally and transferred to the optimization platform, serving as input to guide material synthesis pathways. This integrated system operates in an AI-driven laboratory, enabling real-time material characterization and providing immediate feedback to optimize the synthesis process.

Additionally, we have launched a website at https://xqueryer.caobin.asia/ for real-time PXRD structure identification across multiple sites and platforms.

## CONCLUSIONS AND FUTURE WORK

We present a model for single-phase identification across chemical systems. A simulation pipeline generates high-fidelity data, enabling XQueryer to achieve state-of-the-art accuracy in structure identification. Integrated with diffractometers, it supports efficient workflows for synthesis, characterization and optimization in AI-driven laboratories. Still, several aspects need further development.

(i) *Multi-phase identification:* XQueryer is currently limited to single-phase PXRD patterns. Extending the framework to multi-phase systems is an important next step.
(ii) *Database coverage:* practical performance depends on the completeness of the crystal database. High-quality, system-specific datasets are essential to further improve accuracy.
(iii) *Integration with refinement:* phase identification is closely linked to subsequent structure refinement, which is critical for determining final crystal structures.
(iv) *Additional diversity factors:* XQueryer currently accounts for 12 diversity factors that influence diffraction patterns. However, additional realistic effects, such as twin boundaries, impurities and dislocations, are not yet fully addressed. Incorporating representative experimental data may help mitigate these challenges. In thin-film data, strong orientation can cause peak disappearance and large intensity fluctuations. AI-based strategies tailored for highly oriented, non-powder samples remain to be developed.

## METHODS

### PXRD simulation

The crucial simulation strategy is detailed below. The diffraction vector $\mathbf {Q}$ is defined as


(1) \begin{equation*}
|\mathbf {Q}| = \frac{2\pi \sin {\theta }}{\lambda },
\end{equation*}

where $\lambda$ is the wavelength of the scattered X-ray and $\theta =2\theta /2$, with $2\theta$ being the angle between the incident and diffracted beams. In three dimensions, scattering occurs only at a discrete set of reciprocal vectors, $\mathbf {K}$, forming the reciprocal lattice,


(2) \begin{equation*}
\mathbf {K} = h\mathbf {a}^{*} + k\mathbf {b}^{*} + l\mathbf {c}^{*},
\end{equation*}

where $\mathbf {a}^{*}$, $\mathbf {b}^{*}$ and $\mathbf {c}^{*}$ are the reciprocal lattice vectors, and $h$, $k$ and $l$ are constants. The diffraction condition is defined as


(3) \begin{equation*}
\mathbf {Q}= \mathbf {K}. \end{equation*}

The diffraction intensity $I$ on each diffraction vector $\mathbf {Q}$ for a single phase is determined by


(4) \begin{equation*}
I(\mathbf {Q}) = SF^{*}F\varnothing LPOD + I^{\text{BG}},
\end{equation*}

where $S$ denotes the scale factor, $F$ is the structure factor, $F^{*}$ is the complex conjugate of $F$, $\varnothing$ is the profile function, $L$ is the Lorentz-polarization factor, $P$ is the multiplicity, $O$ is the preferred orientation factor, $D$ is the Debye–Waller factor and $I^{\text{BG}}$ is the background intensity.

The structure factor is computed as


(5) \begin{equation*}
F = \sum _{j=1}^N f_j e^{i \mathbf {Q} \cdot \mathbf {R}},
\end{equation*}

where $f_j$ is the form factor [47] in XRD, $\mathbf {R}$ is the lattice coordinate of atom $j$ and $N$ is the total number of atoms in a lattice cell.

The Lorentz-polarization $L$ is given by


(6) \begin{equation*}
L = \frac{1 + \cos ^2{2\theta }}{\sin ^2{\theta }\cos {\theta }},
\end{equation*}

where $\theta =\arcsin\! {({\lambda |\mathbf {Q}|}/{2\pi })}$. The multiplicity $P$ is determined by counting the number of diffraction vectors present within an Ewald diffraction sphere. The Debye–Waller factor $D$ is given by


(7) \begin{equation*}
D = e^{-2M},
\end{equation*}

where


\begin{equation*}
M = \frac{6h^2 T}{m k \Theta ^2}\bigg [\phi \bigg (\frac{\Theta }{T}\bigg ) + \frac{\Theta }{4T}\bigg ] (\sin ^2{\theta }) \lambda ^{-1}.
\end{equation*}

Here $h$ is Planck’s constant, $m$ is the atom mass, $k$ is Boltzmann constant, $\Theta$ is the average characteristic temperature, $T$ is the absolute temperature and $\phi (\Theta /T)$ is the Debye function.

The profile function ($\varnothing$) is modeled by convolving various factors, including diffraction, detector geometry and noise factors, as shown in Fig. 2(b). The simulated peaks are given by


(8) \begin{equation*}
y(x) = W \otimes G \otimes S.
\end{equation*}

Here, ‘$ \otimes$’ denotes the convolution process, and $ W$, $ G$ and $ S$ represent the contributions to the observed XRD pattern from diffraction emission, instrumental factors and the noise mixture, respectively. Contribution $ S$ is modeled as a Gaussian peak.

Contribution $ W$ is a Voigt function [48],


(9) \begin{eqnarray*}
W &=& \frac{1}{\sigma \sqrt{2\pi }} \int _{-\infty }^{\infty } \bigg [\frac{\gamma }{(2\theta -t)^2 + \gamma ^2}\bigg ]\nonumber\\
&&\times \exp\! \bigg (-\frac{(2\theta -t)^2}{2\sigma ^2}\bigg )dt.
\end{eqnarray*}

Peak broadening is correlated with the full width at half maximum ($\Gamma$), where $2\gamma = 2\sqrt{2\ln {2}}\sigma = \Gamma$ [49]. Here $\Gamma$ is calculated using Scherrer’s equation [50] related to finite grain size.

The geometrical factor [51] $ G$ accounts for the actual dimensions of the detectors and the powder specimen. It is defined as


(10) \begin{equation*}
G(2\alpha , 2\theta ) = \frac{\delta I(2\theta )}{\delta (2\alpha )} \, d(2\alpha ),
\end{equation*}

(11) \begin{equation*}
G(2\alpha , 2\theta ) = \frac{L}{4HSh \cos (2\alpha )} \int dz,
\end{equation*}

where $ 2\alpha$ represents the Bragg angle and $ L$ denotes the distance between the specimen and the detector. The detector is slit shaped, with a height of $ 2H$, and the sample has a height of $ 2S$. See Section S4.1 for more details.

Earlier studies primarily relied on widely used software tools such as Pymatgen [52], FullProf [53] and GSAS-II [54] to simulate diffraction patterns for training purposes. One such early attempt by Lee *et al.* [15] involved training models on simulated data, where factors such as peak profiles, peak positions, background noise, white noise, multiplicity and polarization corrections were incorporated. These parameters are typically deconvoluted during the XRD refinement process. In contrast, our novel simulation tool captures more complex interactions among these factors. It further accounts for additional physical effects, including detector geometry, sample grazing incidence and temperature-induced peak broadening, thereby significantly enhancing the fidelity of the simulated data.

However, models trained solely on simulated data often exhibit performance degradation when evaluated on real experimental datasets. Addressing this challenge requires efforts from at least two complementary directions. First, the development of higher-fidelity simulation tools is essential; to this end, we have open-sourced our simulation code to support broader advancement. Second, the creation of a large-scale, real-world powder XRD database [11] is critical. Such a resource would offer a comprehensive benchmark for assessing model performance and would facilitate the application of techniques such as transfer learning and contrastive learning to accelerate model improvement.

### XQueryer architecture

In XQueryer, PXRD data pass through three functional blocks: the FFT block (Fig. 3(b)), the convolutional block (Fig. 3(c)) and the cross-attention block (Fig. 3(d)). Herein, we applied an FFT-based block to filter PXRD signals in the frequency domain by removing 30%, 60% and 90% of the high-frequency components. This design aims to reduce noise and ease the training burden by suppressing potentially irrelevant fine-grained fluctuations. However, applying fixed cutoff ratios may restrict the model’s ability to adapt to varying data characteristics. A more flexible approach would be to incorporate a learnable gating mechanism that dynamically determines the optimal frequency filtering ratio based on input features. Such a strategy could adapt to variations in peak density, signal-to-noise ratio or structural complexity. In the present work, the FFT block creates multiple levels of coarse-grained perception. The filtered signals are then remapped to reciprocal space via an inverse FFT. They are processed through a no-pooling CNN block, where one-dimensional convolutions with kernel sizes of 16, 32, 64, 128, 256 and 512 capture various levels of coarse graining in reciprocal space.

The FFT block consists of three steps: transform, truncate and reverse. Specifically, for an input sequence $X_n$ containing $N$ reciprocal vectors ($0 \le n \le N - 1$), the FFT block transforms it into frequency space using a 1D FFT:


(12) \begin{equation*}
X_{Rk} = \text{FFT}(X_n).
\end{equation*}

The FFT operates by converting $X_n$ into its conjugate frequency space using the formula


(13) \begin{eqnarray*}
X_{Rk}[k] &=& \sum _{n=0}^{N-1} X_n[n] e^{-j (2\pi /N)kn},\nonumber\\
&&\qquad 0 \le k \le N - 1.
\end{eqnarray*}

Notably, the FFT is applied independently across all dimensions of $X_n$.

In the truncation step, the high-frequency tail of $X_{Rk}$ is removed. The FFT decomposes mixed signals into their frequency components. A high-degree filter reduces scattering and peak overlapping but may obscure fine peak details. Conversely, retaining too many frequencies preserves details but diminishes coarse granularity. To address this trade-off, a hyperparameter $r \in (0, 1)$ determines the proportion of frequency components retained. Consequently, the length of $X_{Rk}$ is reduced from $N$ to $\lceil rN \rceil$ (where $r$ = 0.7, 0.4 or 0.1), enabling multiple levels of coarse-grained filtering.

Finally, the inverse FFT reconstructs the PXRD pattern:


(14) \begin{eqnarray*}
X_n[n] &=& \frac{1}{rN} \sum _{k=0}^{rN-1} X_{Rk}[k] e^{j (2\pi /rN)kn}, \nonumber\\
&&\qquad 0 \le n \le N - 1.
\end{eqnarray*}

The general operation of the FFT block can be expressed as


(15) \begin{eqnarray*}
X_{\text{ft}} = {\bf IFFT}\lbrace {\bf filter}[{\bf FFT}(X)]\rbrace ,
\end{eqnarray*}

where $X$ is the input PXRD pattern.

The output sequence $X_{\text{ft}}$ is passed through a 1D CNN layer:


(16) \begin{equation*}
X_{\text{cnn}} = {\bf CNN}(X_{\text{ft}}).
\end{equation*}

Here, the convolutional kernel traverses the input sequence, performing operations to extract relevant peak shape features such as broadening and asymmetry. The CNN also captures angular dependencies by recognizing patterns occurring at different diffraction angles.

The use of multiple kernel sizes (16, 32, 64, 128, 256 and 512) provides varying receptive fields. Larger kernels are sensitive to long-term angular dependencies, while smaller kernels focus on short-term dependencies. This diversity ensures comprehensive feature extraction across the PXRD data.

The processed tensors $X_{\rm cnn}$ are concatenated into a 3D tensor and passed to the multi-head cross-attention blocks [55], where feature computations are performed as


(17) \begin{eqnarray*}
&&Q= {\bf MLP}_Q(E),\quad K= {\bf MLP}_K(X_{\rm cnn}),\nonumber\\
&& \quad V= {\bf MLP}_V(X_{\rm cnn}),
\end{eqnarray*}

(18) \begin{equation*}
\text{Attention}(Q, K, V) = {\bf softmax}\bigg (\frac{QK^T}{\sqrt{d_k}}\bigg )V,
\end{equation*}

where $E$ denotes the chemical element embeddings obtained through CGCNN atom embeddings [34]. The $\text{MLP}_Q$, $\text{MLP}_K$ and $\text{MLP}_V$ are distinct multi-layer perceptrons that project $E$ and $X_{\rm cnn}$ into $Q$, $K$ and $V$, respectively.

The aggregated feature from the first three functional blocks is mapped to a crystal type by the classification head. While the current pipeline is designed for universal single-phase identification across general chemical systems, several enhancements can enable multi-phase recognition in specific systems. For example, a multi-label classification scheme can be used to predict the presence of multiple phases simultaneously. Alternatively, a mixture density network can model varying phase contributions. These approaches would allow the model not only to detect multi-phase compositions, but also to estimate their relative proportions, thereby extending its applicability.

### Hyperparameters

The hyperparameters are used across all experiments: batch size 128, learning rate $2.5\times 10^{-4}$. All models are trained for 100 epochs with an early stopping patience of 5. We use the cross-entropy function to measure the loss between prediction and the ground truth. We use accuracy, macro F1 score, macro precision, and macro recall as metrics to measure the performance of the models. All models are implemented based on the Pytorch [56] library, trained on GeForce RTX 3090 GPU. Since JADE, as a search-match method, is a training-free model, only prediction accuracy is recorded as the evaluation metric.

## Supplementary Material

nwaf421_Supplemental_File

## Data Availability

All data and code supporting this study are openly available on the XQueryer homepage at https://github.com/Bin-Cao/XQueryer.
