# Supplementary material for: XQueryer: an intelligent crystal structure identifier for powder X-ray diffraction
Source: Natl Sci Rev. 2025 Sep 30;12(12):nwaf421. doi: 10.1093/nsr/nwaf421 (PMC12641137; doi:10.1093/nsr/nwaf421)
Supplement: nwaf421_Supplemental_File [file nwaf421_supplemental_file.pdf]

Supporting Materials of
XQueryer: an intelligent crystal structure identifier for powder X-ray diffraction

Bin Cao[1,2], Zinan Zheng[3], Yang Liu[3,5], Longhan Zhang[1,2], Lawrence W-Y Wong[4], LuTao Weng[1,4], Jia Li[1,3,5*], Haoxiang Li[1,2*], Tong-Yi Zhang[6,1,2*]

[1]Guangzhou Municipal Key Laboratory of Materials Informatics, Hong Kong University of Science and Technology (Guangzhou), Guangzhou, 511400, China.
[2]Advanced Materials Thrust, Hong Kong University of Science and Technology (Guangzhou), Guangzhou, 511400, China.
[3]Data Science and Analytics Thrust, Hong Kong University of Science and Technology (Guangzhou), Guangzhou, 511400, China.
[4]Material Characterization and Preparation Facility, Hong Kong University of Science and Technology (Guangzhou), Guangzhou, 511400, China.
[5] Hong Kong University of Science and Technology, Hong Kong, China.
[6]Materials Genome Institute, Shanghai Frontier Science Center of Mechanoinformatics, and Center for Integrated Circuits and Advanced Display Materials, Shanghai University, Shanghai, 200444,China.

https://www.youtube.com/watch?v=OYPoh7K5uM0.

*Corresponding author(s). E-mail(s): jialee@ust.hk; haoxiangli@hkust-gz.edu.cn; mezhangt@hkust-gz.edu.cn;

1 CONTEXTS

1.1 Section 2 : TABLES

- **Supplemental Table 1**: Detailed ablation experiments of the XQueryer model.
- **Supplemental Table 2**: The performance of baselines with and without the FFT block.
- **Supplemental Table 3**: The performance of baselines with and without the FFT block on broad and narrow peak distributions.
- **Supplemental Table 4**: Summaries of existing powder XRD datasets.

1.2 Section 3 : FIGURES

- **Supplemental Figure 1** gives the accuracy of structure identification, crystal system and space group classification across different models on simulated and experimental test data
- **Supplemental Figures 2 and 3** compare the experimental results for Mn_2O_3, RuO_2, NaCl, $PbSO_4$, $Ba_2Tb_2Co_2O_{11}$, $LiCO_3$, and $LiNiO_2$ with the simulated patterns. The results demonstrate excellent agreement under the optimized simulation parameters.
- **Supplemental Figure 4** : The total number of MP crystals, the number correctly identified by XQueryer, and the identification accuracy rate across **A** the seven crystal systems and **B** the ten space groups, showing the ten most prevalent groups.

1.3 Section 4 : ADDITIONAL BACKGROUND

- **PXRD simulation setting, section 4.1**
- **In-library and Out-of-library tasks, section 4.2**
- **Search-Match approach, section 4.3**
- **Whole powder pattern fitting and refinement, section 4.4**
- **RUFF Data Processing, section 4.5**
- **Hardware-Software Integration Details 4.6**
- **Crystal Structure Determination via PXRD, section 4.7**
- **Model Architecture, section 4.8**

2 TABLES

Table 1. Detailed ablation experiments of the XQueryer model.

	FFT Block		CNN Block		CA Block	
	with	without	with	without	with	without
Accuracy	**0.705**	0.601	**0.705**	0.535	**0.705**	0.527
	Filter Ratio			Kernel Size		
	30%	30/60%	30/60/90%	16-256	16-512	16-1024
Accuracy	0.583	0.627	**0.705**	0.651	**0.705**	0.688
	Number of Cross-attention Layers					
	2	3	4	5	6	7
Accuracy	0.631	0.682	**0.705**	0.702	0.681	0.703

Table 2. The performance of baselines with and without the FFT block.

Structure Identification												
Baselines					Simulation				RRUFF			
**Model**	**Conv.**	**Pooling**	**Ensemble**	**Ref.**	**Accuracy**	**F1**	**Precision**	**Recall**	**Accuracy**	**F1**	**Precision**	**Recall**
CNN1	6	MaxPool	✓	[1]	0.001 $\downarrow_{0.004}$	0.000 $\downarrow_{0.001}$	0.000 $\downarrow_{0.001}$	0.001 $\downarrow_{0.004}$	0.001 $\downarrow_{0.002}$	0.000 $\downarrow_{0.001}$	0.000 $\downarrow_{0.001}$	0.001 $\downarrow_{0.001}$
CNN2	4	MaxPool	×	[2]	0.251 $\uparrow_{0.125}$	0.231 $\uparrow_{0.120}$	0.248 $\uparrow_{0.125}$	0.251 $\uparrow_{0.125}$	0.202 $\uparrow_{0.068}$	0.118 $\uparrow_{0.044}$	0.117 $\uparrow_{0.044}$	0.120 $\uparrow_{0.044}$
CNN3	3	None	×	[3]	0.330 $\uparrow_{0.150}$	0.309 $\uparrow_{0.147}$	0.325 $\uparrow_{0.154}$	0.330 $\uparrow_{0.150}$	0.271 $\uparrow_{0.116}$	0.168 $\uparrow_{0.080}$	0.167 $\uparrow_{0.079}$	0.169 $\uparrow_{0.079}$
**LSTM**					0.002 $\downarrow_{0.104}$	0.001 $\downarrow_{0.078}$	0.001 $\downarrow_{0.077}$	0.002 $\downarrow_{0.104}$	0.004 $\downarrow_{0.120}$	0.002 $\downarrow_{0.065}$	0.002 $\downarrow_{0.065}$	0.002 $\downarrow_{0.067}$
**GRU**					0.046 $\downarrow_{0.069}$	0.031 $\downarrow_{0.051}$	0.030 $\downarrow_{0.050}$	0.046 $\downarrow_{0.069}$	0.035 $\downarrow_{0.049}$	0.018 $\downarrow_{0.026}$	0.018 $\downarrow_{0.026}$	0.019 $\downarrow_{0.027}$
**Bidirectional-LSTM**					0.094 $\downarrow_{0.221}$	0.074 $\downarrow_{0.210}$	0.074 $\downarrow_{0.222}$	0.094 $\downarrow_{0.221}$	0.075 $\downarrow_{0.195}$	0.040 $\downarrow_{0.120}$	0.040 $\downarrow_{0.119}$	0.041 $\downarrow_{0.121}$
**Bidirectional-GRU**					0.269 $\downarrow_{0.147}$	0.234 $\downarrow_{0.147}$	0.242 $\downarrow_{0.155}$	0.269 $\downarrow_{0.147}$	0.209 $\downarrow_{0.088}$	0.119 $\downarrow_{0.060}$	0.118 $\downarrow_{0.060}$	0.121 $\downarrow_{0.062}$
**SegRNN**					0.419 $\uparrow_{0.129}$	0.380 $\uparrow_{0.124}$	0.405 $\uparrow_{0.125}$	0.419 $\uparrow_{0.129}$	0.313 $\uparrow_{0.100}$	0.191 $\uparrow_{0.068}$	0.190 $\uparrow_{0.069}$	0.195 $\uparrow_{0.069}$
**iTransformer**					0.340 $\uparrow_{0.019}$	0.313 $\uparrow_{0.019}$	0.331 $\uparrow_{0.021}$	0.340 $\uparrow_{0.019}$	0.241 $\uparrow_{0.012}$	0.141 $\uparrow_{0.009}$	0.139 $\uparrow_{0.008}$	0.143 $\uparrow_{0.009}$
**PatchTST**					0.239 $\uparrow_{0.070}$	0.209 $\uparrow_{0.064}$	0.225 $\uparrow_{0.065}$	0.239 $\uparrow_{0.070}$	0.159 $\uparrow_{0.038}$	0.090 $\uparrow_{0.023}$	0.089 $\uparrow_{0.023}$	0.091 $\uparrow_{0.022}$

Table 3. The performance of baselines With and Without FFT block on broad and narrow peak distributions.

Structure Identification												
Baselines					Broad				Narrow			
**Model**	**Conv.**	**Pooling**	**Ensemble**	**Ref.**	**Accuracy**	**F1**	**Precision**	**Recall**	**Accuracy**	**F1**	**Precision**	**Recall**
CNN1	6	MaxPool	✓	[1]	0.027 $\downarrow_{0.012}$	0.006 $\downarrow_{0.002}$	0.004 $\downarrow_{0.001}$	0.027 $\downarrow_{0.012}$	0.062 $\downarrow_{0.002}$	0.017 $\downarrow_{0.002}$	0.011 $\downarrow_{0.001}$	0.062 $\downarrow_{0.002}$
CNN2	4	MaxPool	×	[2]	0.287 $\uparrow_{0.025}$	0.262 $\uparrow_{0.023}$	0.281 $\uparrow_{0.021}$	0.287 $\uparrow_{0.025}$	0.261 $\uparrow_{0.010}$	0.236 $\uparrow_{0.09}$	0.257 $\uparrow_{0.006}$	0.261 $\uparrow_{0.010}$
CNN3	3	None	×	[3]	0.386 $\uparrow_{0.105}$	0.358 $\uparrow_{0.101}$	0.376 $\uparrow_{0.106}$	0.386 $\uparrow_{0.105}$	0.277 $\uparrow_{0.036}$	0.260 $\uparrow_{0.037}$	0.273 $\uparrow_{0.037}$	0.277 $\uparrow_{0.036}$
**LSTM**					0.079 $\uparrow_{0.051}$	0.067 $\uparrow_{0.052}$	0.070 $\uparrow_{0.056}$	0.079 $\uparrow_{0.051}$	0.070 $\uparrow_{0.067}$	0.057 $\uparrow_{0.056}$	0.058 $\uparrow_{0.057}$	0.070 $\uparrow_{0.067}$
**GRU**					0.093 $\uparrow_{0.034}$	0.076 $\uparrow_{0.036}$	0.079 $\uparrow_{0.041}$	0.093 $\downarrow_{0.034}$	0.064 $\downarrow_{0.001}$	0.048 $\uparrow_{0.006}$	0.048 $\uparrow_{0.009}$	0.064 $\downarrow_{0.001}$
**Bidirectional-LSTM**					0.049 $\downarrow_{0.187}$	0.034 $\downarrow_{0.166}$	0.032 $\downarrow_{0.175}$	0.049 $\downarrow_{0.187}$	0.001 $\downarrow_{0.001}$	0.001 $\downarrow_{0.001}$	0.001 $\downarrow_{0.001}$	0.001 $\downarrow_{0.002}$
**Bidirectional-GRU**					0.221 $\downarrow_{0.076}$	0.188 $\downarrow_{0.174}$	0.193 $\downarrow_{0.078}$	0.221 $\downarrow_{0.076}$	0.116 $\downarrow_{0.17}$	0.091 $\downarrow_{0.154}$	0.090 $\downarrow_{0.042}$	0.116 $\downarrow_{0.166}$
**SegRNN**					0.367 $\uparrow_{0.106}$	0.331 $\uparrow_{0.105}$	0.353 $\uparrow_{0.107}$	0.367 $\uparrow_{0.106}$	0.386 $\uparrow_{0.140}$	0.349 $\uparrow_{0.135}$	0.374 $\uparrow_{0.141}$	0.386 $\uparrow_{0.140}$
**iTransformer**					0.307 $\uparrow_{0.019}$	0.279 $\uparrow_{0.019}$	0.294 $\uparrow_{0.021}$	0.307 $\uparrow_{0.019}$	0.348 $\uparrow_{0.016}$	0.317 $\uparrow_{0.017}$	0.335 $\uparrow_{0.018}$	0.348 $\uparrow_{0.016}$
**PatchTST**					0.204 $\uparrow_{0.039}$	0.176 $\uparrow_{0.024}$	0.189 $\uparrow_{0.022}$	0.204 $\uparrow_{0.029}$	0.159 $\uparrow_{0.038}$	0.090 $\uparrow_{0.023}$	0.089 $\uparrow_{0.023}$	0.091 $\uparrow_{0.022}$

Table 4. Summaries of existing powder XRD datasets. ICSD refers to the commercial Inorganic Crystal Structure Database. MP denotes the open-sourced Material Project.

Dataset	XRD Pattern	Structure	Open Access	Simulated	Crystal Source	Year
**RRUFF** [4]	3,002	3,002	✓	×	-	2015
**XRDSP** [5]	169,536	169,536	✓	✓	**ICSD**	2020
**CNN** [6]	1,785,405	170	×	✓	**ICSD**	2020
**PQNet** [7]	250,000	1	✓	✓	**ICSD**	2021
**XRDAutoAnalyzer** [1]	38,250	150	✓	✓	**ICSD**	2021
**XRDIsAllYouNeed** [8]	328,503	189,476&139,02	×	✓	**ICSD&MP**	2022
**AdvancedXRDAnalysis** [9]	29,569,650	197,131	×	✓	**ICSD**	2023
**CrySTINet** [10]	100	100	✓	✓	**ICSD**	2024
**CPICANN** [11]	692,190	23,073	✓	✓	**COD**	2024
**SimXRD**[12]	4,065,346	119,569	✓	✓	**MP**	2024

3 FIGURES

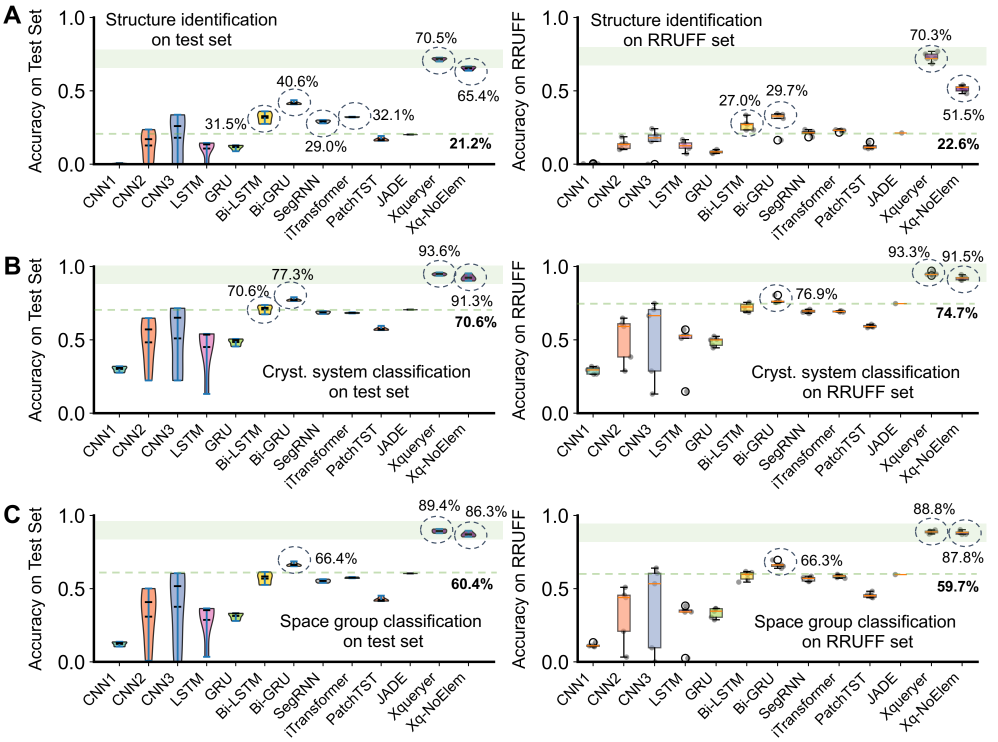

Figure 1. The performance of models across three tasks: **A** Structure identification, **B** Crystal system classification, and **C** Space group classification. The results are tested on 200,630 simulated PXRD test data and the RRUFF experimental data. The green dotted line represents the performance of the conventional search-match method.

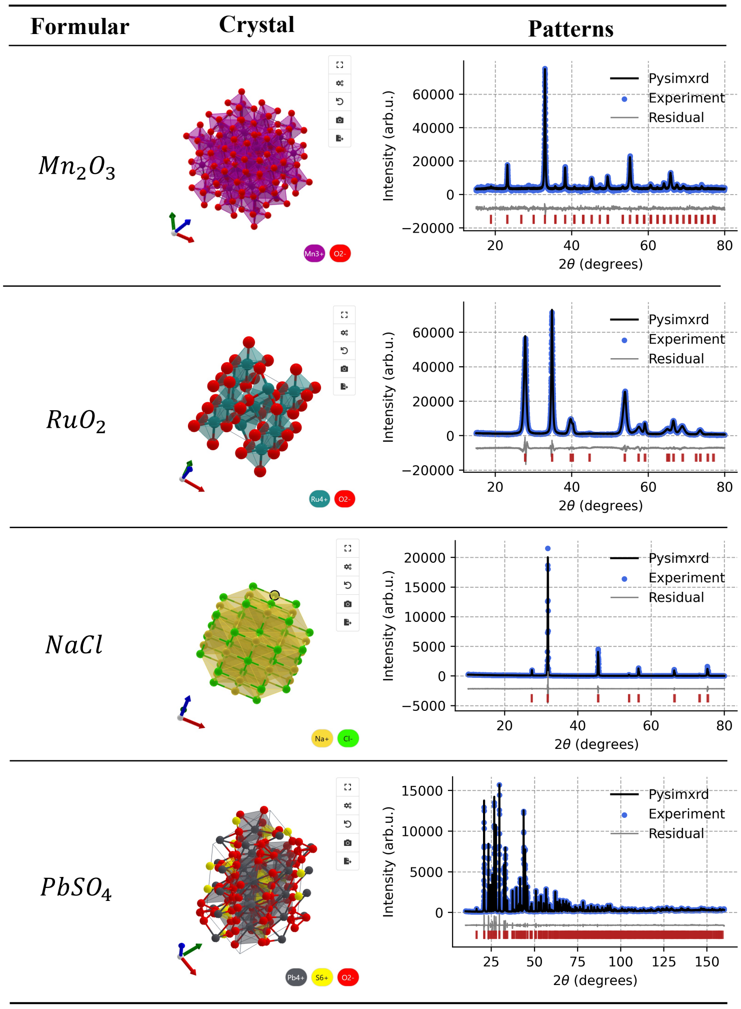

Figure 2. (Mn_2O_3, RuO_2, NaCl, $PbSO_4$) The case studies of our simulation method: a comparison between the experimental PXRD pattern and the simulated pattern shows excellent consistency under carefully optimized simulation parameters

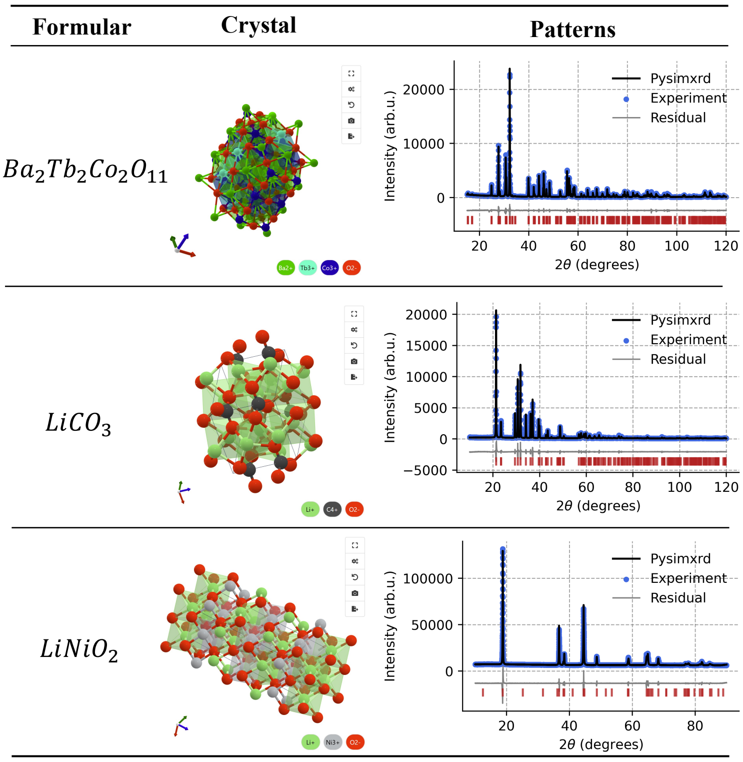

Figure 3. ($Ba_2Tb_2Co_2O_{11}$, $LiCO_3$, and $LiNiO_2$) The case studies of our simulation method: a comparison between the experimental PXRD pattern and the simulated pattern shows excellent consistency under carefully optimized simulation parameters

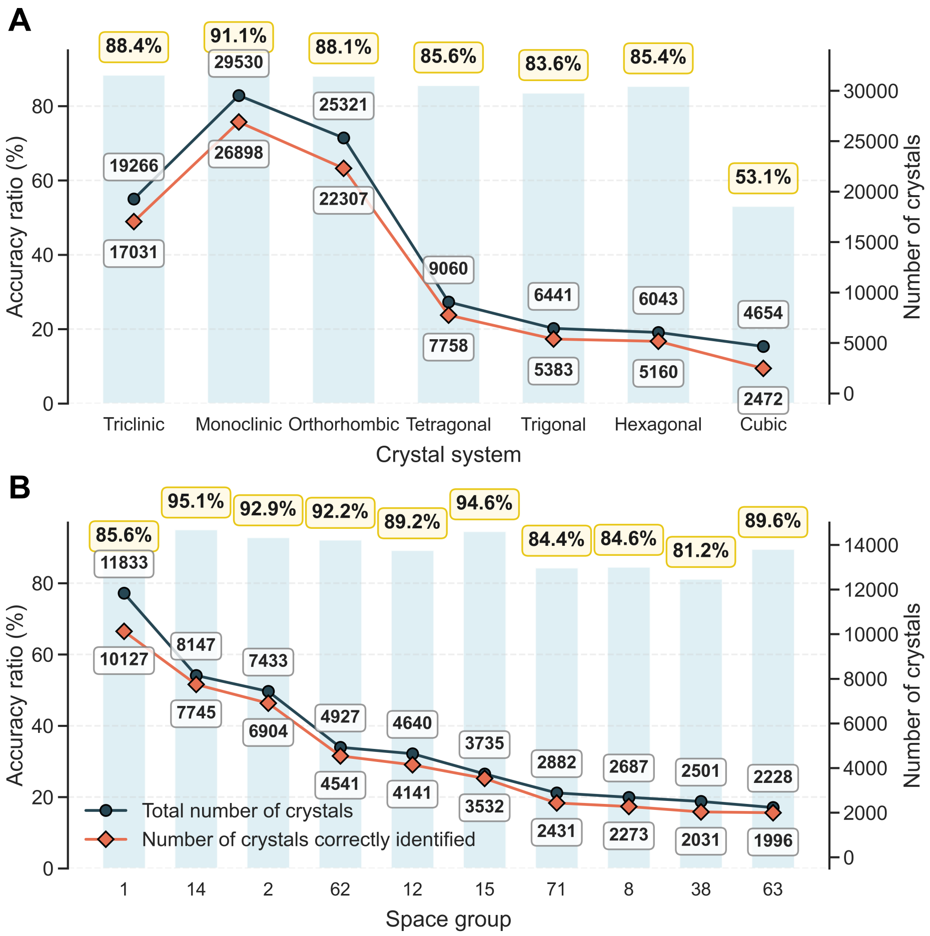

Figure 4. The total number of MP crystals, the number correctly identified by XQueryer, and the identification accuracy rate across **A** the seven crystal systems and **B** the ten space groups, showing the ten most prevalent groups.

4 ADDITIONAL BACKGROUND

4.1 PXRD simulation setting

For each crystal, we account for diverse conditions using the Pysimxrd [13]. The simulation allows adjustment of the following parameters:

- **Grain Size:** The average grain size is set within the range of 10 to 150 nm.
- **Orientation:** An ideal powder specimen is assumed to have no preferred orientation. However, this condition is practically unattainable. Orientation effects arise due to the uneven distribution of small grains in the incident beam, causing a non-uniform distribution of reciprocal sites across the reciprocal spheres of the powder sample. To simulate these effects, the intensity is perturbed within a 30% range based on the ideal simulation to mimic orientation randomness.
- **Thermal Vibration:** Temperature is converted into the kinetic energy of atoms. Atomic vibrations are simulated by allowing atoms to deviate from their average positions within a range of 0.01 to 0.1 Å.
- **Internal Stress:** Internal stress is modeled by applying elastic deformation to the recorded lattice constant, with variations up to 10%.
- **Instrument Zero Shifting:** Zero shifting is simulated by randomly translating the 2θ value within a range of -2° to 2°.
- **Instrumental Noise:** Gaussian white noise, with an intensity of 10%, is added across the entire pattern to represent instrumental noise.
- **Scattering:** A sixth-order polynomial function is used to simulate background distribution. This accounts for effects such as Compton scattering, fluorescence, and multiple scattering. The simulated background is then incorporated into the XRD pattern with a 10% contribution.
- **Detector and Sample Heights:** The detector height is set within the range of 5 to 15 mm, while the sample height is specified between 1 and 8 mm.

4.2 In-library and Out-of-library tasks

In-library identification is a fundamental task in crystallography that aims to identify crystal types based on XRD patterns measured in various environments. Since 1938, crystallographers have been documenting all discovered structures and archiving them as Powder Diffraction Files (PDFs). By comparing and retrieving these PDFs, researchers can determine the structures of studied materials. With advancements in computing, numerous software programs have been developed to assist in the search-match process.

A related concept is out-of-library identification, which involves discovering previously unknown structures that lack any recorded information. This process relies on symmetry identification to determine the basic space group and subsequent refinement processes. The crystal system is typically determined by its physical properties, such as electrical conductivity, optical behavior, and thermal characteristics. The space group is then identified through extinction effects. Next, the ideal chemical formula is derived based on the estimated number of atoms and Wyckoff positions. Validation follows through refinement and site optimization.

4.3 Search-Match approach

The predominant method for in-library structure identification in X-ray powder diffraction patterns is the search-match approach, which involves three key steps. First, (d, I) values—representing interplanar spacing (d) and peak intensity (I)—are extracted from the diffraction pattern. Next, potential phases are identified by searching diffraction databases for matching d values, such as those in the Hanawalt indexes [14]. Candidate phases are then compared to the pattern's (d, I) values using a scoring system, helping to select the best match. This process continues until satisfactory alignment is achieved for most (d, I) values. Despite advances in computer technology enabling novel qualitative methods, the core goal remains to match experimental data with database entries and compute a corresponding score.

In-library query strategies, like the search-match approach, retrieve reference structures for further refinement. These references, based on lattice geometry, provide atomic location information, easing subsequent optimization. However, this method is ineffective for identifying out-of-library structures. In contrast, determining the space group enables the identification of the lattice structure, followed by atomic site optimization during the refinement process. This offers a more general approach for structural measurement.

4.4 Whole powder pattern fitting and refinement

Refinement plays a crucial role in determining crystal structures. It starts with reasonably estimated physical parameters, followed by their optimization. However, without prior structural knowledge, the process turns into trial and error.

Identifying the crystal type begins the process, but its validation relies on the refinement results. The Rietveld method [15, 16] marked a significant advancement in powder diffraction fitting for structure refinement by introducing whole-pattern fitting, replacing the analysis of individual, non-overlapping Bragg peaks. The approach employs profile intensity calculations and a least-squares algorithm for refinement. By minimizing discrepancies between experimental and theoretical profiles, it extracts all structural information. Once the fitting process converges, the parameters in the theoretical model represent the only determinable physical quantities. Subsequently, the Pawley [17] and Le Bail [18] methods, two widely used whole powder pattern fitting techniques, are developed following the Rietveld method.

4.5 RRUFF Data Processing

We downloaded the entire RRUFF dataset in March 2024. The dataset includes an `XY_RAW` folder containing powder X-ray diffraction data, and a `DIF` folder storing crystal structure information. First, we match files from the two folders based on their RRUFF IDs. For each matched pair, we save the diffraction data and corresponding crystal structure in an ASE database file, resulting in a total of 2,088 matched entries.

However, many entries lacked either diffraction data (e.g., 2θ range $< 30°$) or essential crystallographic information such as lattice constants. After filtering out incomplete entries, we obtain 1,003 valid samples. We then label the valid RRUFF entries using MP IDs through a two-level matching strategy:

- **Strict Matching**: The MP entry must have the same elemental composition as the RRUFF sample and lattice constants within a 5% deviation. These matches are labeled as `strict` in the file `matched_pairs.txt`.
- **Relaxed Matching**: The MP entry may differ in elemental composition, but lattice constants must fall within a 1% deviation. These matches are labeled as `relaxed` in the same file.

This process yielded 849 labeled RRUFF entries for in-laboratory structure identification. For space group and crystal system analysis, all 1,003 valid samples are used. The `matched_pairs.txt` file is available in the official XQueryer GitHub repository.

4.6 Hardware-Software Integration Details

We integrate the XQueryer model with the PANalytical Aeris Benchtop X-Ray Diffractometer, achieving a streamlined pipeline for automated powder X-ray diffraction (PXRD) analysis. The full integration process consists of the following steps:

1. **Hardware Setup:** The PANalytical Aeris diffractometer is installed and configured. The system supports third-party integration via file system access or plugin interfaces.
2. **Software Deployment:** The XQueryer model and all required dependencies are installed on the same system (or a connected server). A lightweight monitoring module is deployed to handle directory watching and task automation.
3. **Root Directory Definition:** A fixed directory (e.g., `D:/XRDdata/`) is set as the shared working folder. All output files from the diffractometer (typically in `.xrdml` format) are automatically saved to this location.
4. **Sample Measurement:** Powder samples are mounted on the diffractometer and scanned. Upon scan completion, a new `.xrdml` file is generated in the root directory.
5. **Automatic Trigger:** The monitoring module detects new files in the directory and initiates the processing pipeline. No manual intervention is needed beyond the initial measurement.
6. **Data Parsing and Standardization:** The XQueryer parser reads the `.xrdml` file, extracts the raw 2θ–intensity signal, and applies preprocessing steps (e.g., smoothing, baseline correction, normalization) to convert it into a standardized PXRD pattern.
7. **Structure Identification:** The standardized PXRD pattern is fed into the XQueryer model for phase identification. The model outputs predicted crystal phase along with MP links.
8. **Result Storage:** All results, including text-based identification outputs and optional visual plots, are saved to the same directory as the original `.xrdml` file, ensuring traceability.

This integration supports fully automated phase identification from powder samples. Optional enhancements include batch processing, real-time error logging, and downstream links to visualization or structural analysis tools such as VESTA.

4.7 Crystal Structure Determination via PXRD

Crystal structure determination via PXRD typically involves two main steps:

1. **Initial structure identification:** interpreting the experimental PXRD pattern to propose a plausible (partial or approximate) structure.
2. **Structure refinement:** using the proposed structure as input for further relaxation and optimization to best fit the experimental data.

The first step focuses on identifying a rough structural model, while the second step fine-tunes this model to capture structural details. It is well understood that no two crystals are exactly identical, even for the same compound. For example, NaCl crystals synthesized under different conditions can exhibit structural variations and defects that must be resolved through refinement.

However, refinement cannot begin from scratch. It works by minimizing the differences between the observed PXRD profile and the theoretical pattern calculated from a structural model. Once this fitting process converges, the refined parameters represent the only physically meaningful and experimentally determinable information. Therefore, an accurate initial guess of the crystal structure is essential for successful refinement. This task—*structure identification*—is the focus of this paper.

The purpose of structure identification is to recognize known reference crystals from their PXRD patterns. For instance, identifying that a given pattern belongs to NaCl, without needing to determine precise lattice parameters or defects. For the past few decades, a **search-match strategy** has been widely adopted. By comparing experimental patterns against large crystal structure databases, this approach retrieves candidate structures that best match the observed data and recommends the most similar ones. This process essentially confirms the tested sample based on historical crystallographic knowledge and is referred to as *in-library identification*.

In in-library identification, the effectiveness of the search-matching algorithm depends on how accurately it can retrieve patterns while tolerating variations in real-world physical settings. Since the discovery of truly novel structures is relatively rare, in-library identification remains the mainstream approach in structure determination.

Only when all known structures fail to match does *out-of-library identification* begin. Traditionally, this is a labor-intensive process involving the determination of lattice geometry, ideal chemical composition, and iterative refinement with trial and error. In recent years, generative methods, those that propose structures from scratch, have been increasingly applied in such situations. However, experimental crystal structure determination remains highly challenging, and generation from scratch carries a high risk of producing incorrect structures.

Regardless of the situation, determining a crystal structure, especially a novel one, cannot rely on a single measurement technique. Multimodal determination is often necessary to provide complementary evidence and achieve reliable structural identification. This typically involves integrating data from various sources such as X-ray diffraction (XRD), scanning or transmission electron microscopy (SEM/TEM), and spectroscopic methods like Raman or infrared (IR) spectroscopy. Each technique offers unique structural insights. For example, XRD provides long-range order, electron microscopy reveals local atomic arrangements, and spectroscopy gives information on bonding environments. By cross-validating results across these modalities, ambiguities in phase assignment, symmetry, or atomic positions can be minimized, thereby enhancing both the accuracy and confidence in the final structure.

4.8 Model Architecture

Listing 1. XQueryer architecture

```
XQueryer(
  (conv): ConvModule(
    (conv1): Conv1d(1, 32, kernel_size=17, stride=1, padding=8)
    (bn1): BatchNorm1d(32)
    (act1): ReLU()

    (conv2): Conv1d(1, 32, kernel_size=33, stride=1, padding=16)
    (bn2): BatchNorm1d(32)
    (act2): ReLU()

    (conv3): Conv1d(1, 32, kernel_size=65, stride=1, padding=32)
    (bn3): BatchNorm1d(32)
    (act3): ReLU()

    (conv4): Conv1d(1, 32, kernel_size=129, stride=1, padding=64)
    (bn4): BatchNorm1d(32)
    (act4): ReLU()

    (conv5): Conv1d(1, 32, kernel_size=257, stride=1, padding=128)
    (bn5): BatchNorm1d(32)
    (act5): ReLU()

    (conv6): Conv1d(1, 32, kernel_size=513, stride=1, padding=256)
    (bn6): BatchNorm1d(32)
    (act6): ReLU()
  )

  (encoder): SelfAttnModule(
    (layers): ModuleList(
      (0-2): 3 x CrossAttnLayer(
        (element_map): Sequential(
          (0): Linear(92 -> 2688000)
          (1): Dropout(p=0.5)
          (2): ReLU()
        )
        (cross_attn): MultiheadAttention(3500)
        (linear1): Linear(3500 -> 768)
        (dropout): Dropout(p=0.0)
        (linear2): Linear(768 -> 3500)
        (norm1): LayerNorm(3500)
        (norm2): LayerNorm(3500)
        (dropout1): Dropout(p=0.0)
        (dropout2): Dropout(p=0.0)
      )
    )
  )

  (norm_after): LayerNorm(3500)

  (cls_head): Sequential(
    (0): Linear(3500 -> 2048)
    (1): BatchNorm1d(2048)
    (2): ReLU()
    (3): Dropout(p=0.5)

    (4): Linear(2048 -> 1024)
    (5): BatchNorm1d(1024)
    (6): ReLU()
    (7): Dropout(p=0.5)

    (8): Linear(1024 -> 100315)
  )
)
```

REFERENCE

[1] Szymanski NJ, Bartel CJ, Zeng Y et al. Probabilistic deep learning approach to automate the interpretation of multi-phase diffraction spectra. *Chem Mater* 2021; **33**: 4204–15.

[2] Le NQ, Pekala M, New A et al. Deep learning models to identify common phases across material systems from x-ray diffraction. *J Phys Chem C* 2023; **127**: 21758–67.

[3] Salgado JE, Lerman S, Du Z et al. Automated classification of big x-ray diffraction data using deep learning models. *npj Comput Mater* 2023; **9**: 214.

[4] Lafuente B, Downs RT, Yang H et al. The power of databases: the RRUFF project. *Highlights Mineral Crystallogr* 2015; **1**: 25.

[5] Suzuki Y, Hino H, Hawai T et al. Symmetry prediction and knowledge discovery from x-ray diffraction patterns using an interpretable machine learning approach. *Sci Rep* 2020; **10**: 21790.

[6] Lee JW, Park WB, Lee JH et al. A deep-learning technique for phase identification in multiphase inorganic compounds using synthetic XRD powder patterns. *Nat Commun* 2020; **11**: 86.

[7] Dong H, Butler KT, Matras D et al. A deep convolutional neural network for real-time full profile analysis of big powder diffraction data. *npj Comput Mater* 2021; **7**: 74.

[8] Lee BD, Lee JW, Park WB et al. Powder x-ray diffraction pattern is all you need for machine-learning-based symmetry identification and property prediction. *Adv Intell Syst* 2022; **4**: 2200042.

[9] Lee BD, Lee JW, Ahn J et al. A deep learning approach to powder x-ray diffraction pattern analysis: Addressing generalizability and perturbation issues simultaneously. *Adv Intell Syst* 2023; **5**: 2300140.

[10] Chen L, Wang B, Zhang W et al. Crystal structure assignment for unknown compounds from x-ray diffraction patterns with deep learning. *J Am Chem Soc* 2024; **146**: 8098–109.

[11] Cao B. CPICANN. https://huggingface.co/datasets/caobin/datasetCPICANN (21 May 2024, date last accessed).

[12] Cao B, Liu Y, Zheng Z et al. SimXRD-4M: Big simulated x-ray diffraction data and crystal symmetry classification benchmark. In: *Proc Int Conf Learn Represent* 2025.

[13] Cao B, Liu Y, Zheng Z et al. SimXRD-4M: Big simulated x-ray diffraction data and crystal symmetry classification benchmark. In: *Proc Int Conf Learn Represent* 2025.

[14] University College London. X-ray Powder Diffraction Quality Assurance Tables. http://pd.chem.ucl.ac.uk/pxrd/qa/smtable.htm (date last accessed).

[15] Rietveld H. Line profiles of neutron powder-diffraction peaks for structure refinement. *Acta Crystallogr* 1967; **22**: 151–52.

[16] Rietveld HM. A profile refinement method for nuclear and magnetic structures. *J Appl Crystallogr* 1969; **2**: 65–71.

[17] Pawley G. Unit-cell refinement from powder diffraction scans. *J Appl Crystallogr* 1981; **14**: 357–61.

[18] Le Bail A, Duroy H, Fourquet JL. Ab-initio structure determination of $LiSbWO_6$ by x-ray powder diffraction. *Mater Res Bull* 1988; **23**: 447–52.
